# Harnessing multiplex crRNA enables an amplification-free/CRISPR-Cas12a-based diagnostic methodology for *Nosema bombycis*

**DOI:** 10.1128/spectrum.03014-23

**Published:** 2023-11-28

**Authors:** Huarui Zhang, Huijuan Zhao, Lu Cao, Bin Yu, Junhong Wei, Guoqing Pan, Jialing Bao, Zeyang Zhou

**Affiliations:** 1 State Key Laboratory of Resource Insects, Southwest University, Chongqing, China; 2 Chongqing Key Laboratory of Microsporidia Infection and Control, Southwest University, Chongqing, China; 3 College of Life Science, Chongqing Normal University, Chongqing, China; Chinese Academy of Sciences, Shanghai Institute of Plant Physiology and Ecology, Shanghai, China

**Keywords:** *Nosema bombycis*, CRISPR/Cas12a, multiplex crRNA, amplification-free DNA diagnostic, pathogen detection

## Abstract

*Nosema bombycis* is transmitted horizontally and vertically from infected female moths to progeny eggs, bringing substantial economic losses to sericulture production. Timely and accurate detection of *N. bombycis* can effectively block the spread of silkworm pébrine disease, which is of great significance for its prevention and control. In this study, we established a multiplex-crRNA CRISPR/Cas12a nucleic acid amplification-free detection method for *N. bombycis*.. Specificity and sensitivity assessments confirmed that this method specifically detects *N. bombycis* without cross-reacting with other pathogens. It can detect *N. bombycis* at levels as low as 2 pg of genomic DNA. This method could also provide information on pathogen quantification, which will be important for in-field applications and promotions.

**IMPORTANCE:**

The multiplex-crRNA CRISPR/Cas12a detection method saves hands-on time, reduces the risk of aerosol pollution, and can be directly applied to detecting silkworms infected with *Nosema bombycis*. This study provides a new approach for the inspection and quarantine of silkworm pébrine disease in sericulture and provides a new method for the detection of other pathogens.

## INTRODUCTION

Microsporidia are fungus-like pathogens which are specialized in intracellular parasitism. They have a wide range of hosts and can infect almost all invertebrates and vertebrates (1). *Nosema bombycis* is one of the earliest identified microsporidia, which can cause fatal damage to sericulture production through ingestion and embryo infection. Therefore, *N. bombycis* is the obligated quarantine pathogen in sericulture and serves as the subject and challenge of sericulture research (2). The primary diagnostic techniques for *N. bombycis* involve morphological identification, immunological detection utilizing proteins [e.g., enzyme linked immunosorbent assay (ELISA) (3) and matrix-assisted laser desorption ionization time of flight mass spectrometry (MALDI-TOF-MS) (4)], and molecular biological detection using nucleic acids, with PCR usually employed as a detection method (5).

The CRISPR/Cas system, consisting of clustered regularly interspaced short palindromic repeats and CRISPR-associated proteins, is a defense mechanism commonly found in bacteria and archaea (5 – 7). It works by transcribing specific spacer sequences into small RNA that guides the Cas protein to cut DNA or RNA, thus playing a targeted role (8). Due to its high specificity, programmability, and compatibility with real-time monitoring technology, the CRISPR/Cas system has shown promising potential in the field of molecular diagnosis (9). For instance, CRISPR/Cas13 and CRISPR/Cas12 are both widely used in pathogens and disease detections and diagnosis (10). The CRISPR/Cas13 is RNA-targeting, while the CRISPR/Cas12 is DNA-targeting. Interestingly, the CRISPR/Cas12 has the target DNA-recognition-based collateral cleavage abilities on non-targeted single-stranded DNA (ssDNA) (11). The principle is that Cas12a cleaves the target DNA of 18–25 nt downstream of the protospacer-adjacent motif (PAM) sequence under the guidance of crRNA and simultaneously activates a nonspecific ssDNA incidental cleavage. Researchers integrated the fluorescence reporter and quenching groups into ssDNA based on this collateral cleavage capability. Once it was cleaved, the quenching group was separated from the fluorescence group to generate a fluorescence signal, thus realizing the direct presentation of the cleavage results. It has been applied more and more widely to the detection of various pathogens (12). Researchers have developed many detection methods based on the CRISPR/Cas12a system, such as the DETECTR method combined with RPA technology (13), the HOLMES method combined with PCR technology (14), etc. While combining the CRISPR/Cas system with nucleic acid amplification technology can improve sensitivity and specificity, it also increases the cost of detection due to additional amplification steps. Moreover, this combination usually requires a two-step detection process involving an intermediate transfer between amplification and detection, leading to the potential aerosol pollution of amplified nucleic acid.

Compared with a single crRNA system, multiplex crRNA strategies have been developed in recent years (15 – 17). The principle of this method is designing multiple crRNA complementary to the same target sequence. With the increased number of ssDNA probes being cut in multiplex crRNA systems, the fluorescence signal enhanced without pre-amplification. Based on this premise, our study proposes a method to detect *N. bombycis* by using multiplex crRNA strategies, aiming for an amplification-free CRISPR/Cas12a-based detection system. We evaluate the specificity and sensitivity of this newly developed method and applied in the actual detection of silkworm infected with *N. bombycis*.

## RESULTS

### Design and evaluation of crRNA for rDNA of *N. bombycis*

**Fig 1:**
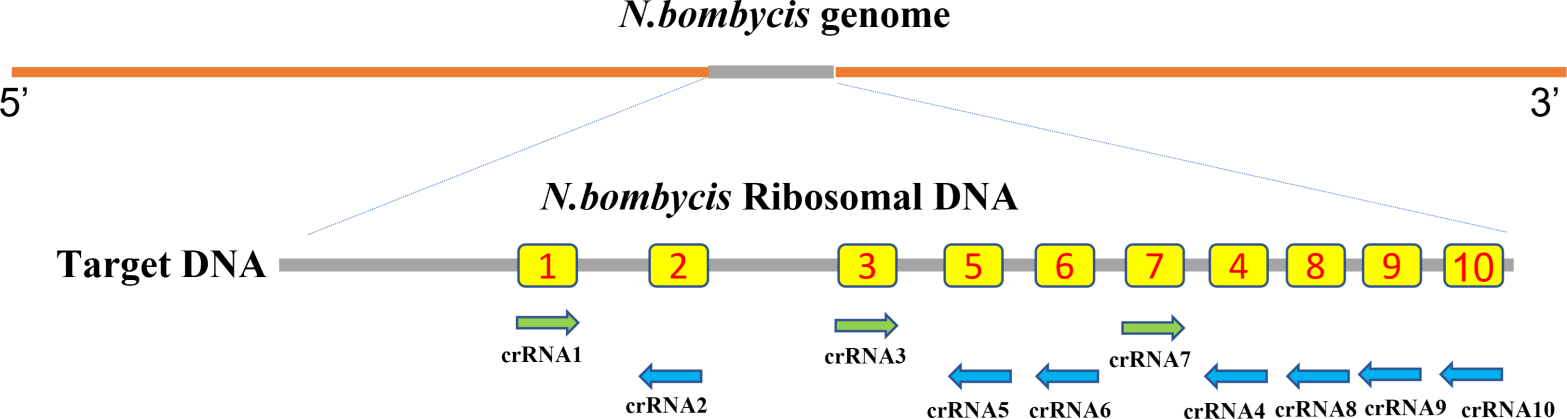
Design of crRNA. Schematic of the *N. bombycis* ribosomal DNA and the corresponding location of each crRNA spacer region.

Using the CRISPR/Cas12a detection system stipulations, 10 crRNA sequences were created, meeting the criteria for eligibility (Fig. 1; Table 1).

**TABLE 1:** Detailed sequence information

	Name	Sequence (5’ to 3’)
CRISPR reaction	crRNA 1	UAAUUUCUACUAAGUGUAGAUGCUGGGGCGGCACAACUGUUAUA
	crRNA 2	UAAUUUCUACUAAGUGUAGAUAUGUAUUAGGAUUCUAACUAUGU
	crRNA 3	UAAUUUCUACUAAGUGUAGAUGUGUGUAUGAUGAUUGAUGCAGU
	crRNA 4	UAAUUUCUACUAAGUGUAGAUGACAGAUGUAGUGAUACAUAUGA
	crRNA 5	UAAUUUCUACUAAGUGUAGAUAACAGAAGCGAAAGCUGUAUACU
	crRNA 6	UAAUUUCUACUAAGUGUAGAUAGAUACCAUUGUAGUUCUAGCAG
	crRNA 7	UAAUUUCUACUAAGUGUAGAUCCAGGUAUAACAUGGUAUAAUAU
	crRNA 8	UAAUUUCUACUAAGUGUAGAUAUAUUUGAACAUGGAAUUGCUAG
	crRNA 9	UAAUUUCUACUAAGUGUAGAUUGAUAUAAGGAGGUUAUAUUGGC
	crRNA 10	UAAUUUCUACUAAGUGUAGAUCAUCAGAUACGGUCAUAUCUACU
	ssDNA reporters	TTTTTTT (5’FAM 3’BHQ1)
PCR	N.b-F	GATCAATAGGATGTCATAACGATG
	N.b-R	GTGGGTTCCAGTGGTTTTCAGTAG

**Fig 2:**
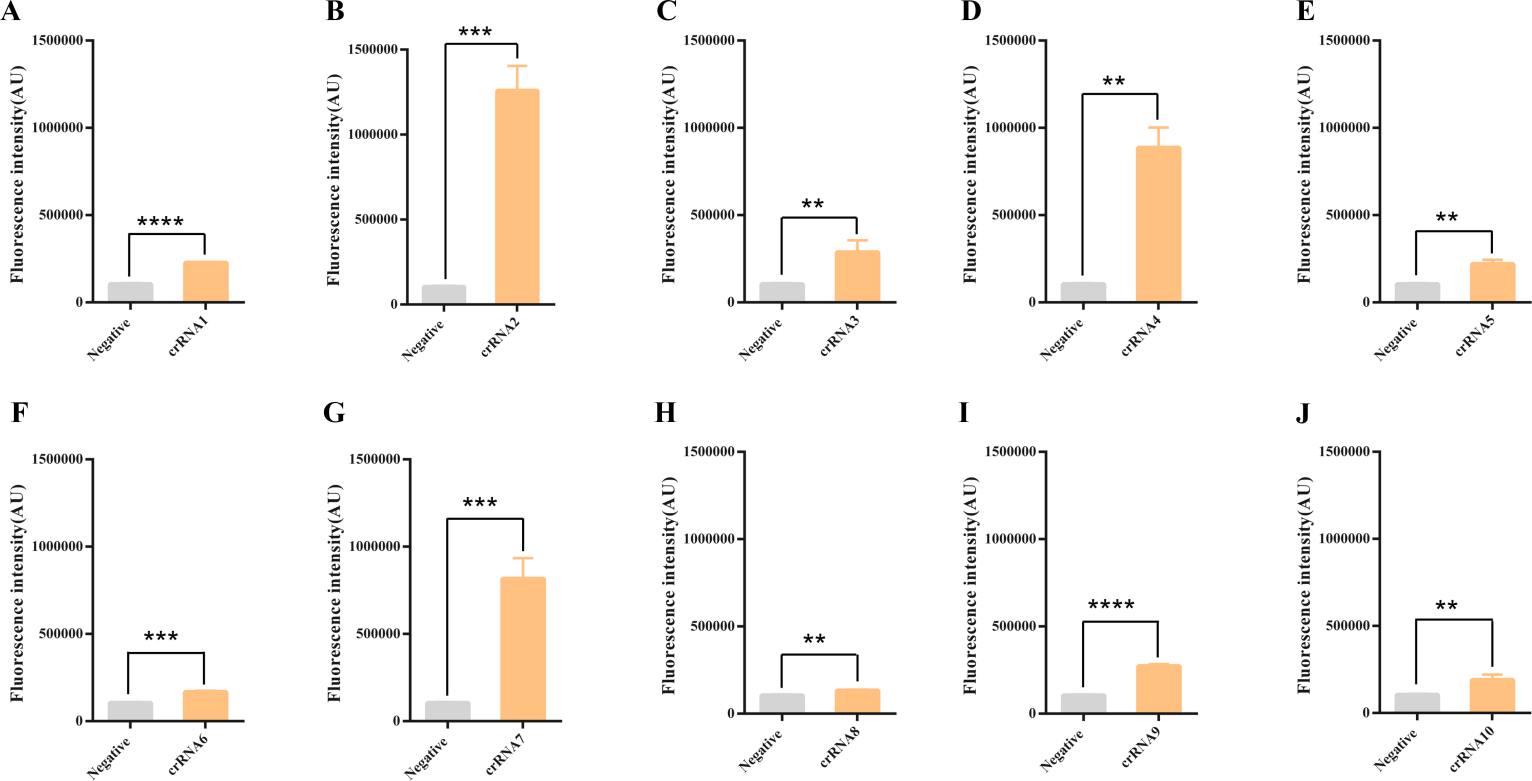
Screening of crRNA. The fluorescence responses of 10 crRNAs (**A–J**) to *N. bombycis* ribosomal DNA were tested in the multiplex-crRNA CRISPR/Cas12a system. The negative control was the detection reaction without adding crRNA. Data were compared by *t* tests. (**P < 0.01, ***P < 0.001, and ****P < 0.0001).

To assess the detection efficiencies of these crRNAs, a negative control was employed utilizing water devoid of nuclease activity instead of a DNA substrate. Each crRNA was individually tested by adding 100 ng of the target DNA substrate (Fig. 2A through J). The study’s findings indicate that crRNA 2, crRNA 4, crRNA 7, and crRNA 3 displayed higher levels of activity compared to the negative control. Notably, crRNA 2 exhibited the highest efficiency in terms of detection.

### Screening of crRNA combinations

**Fig 3:**
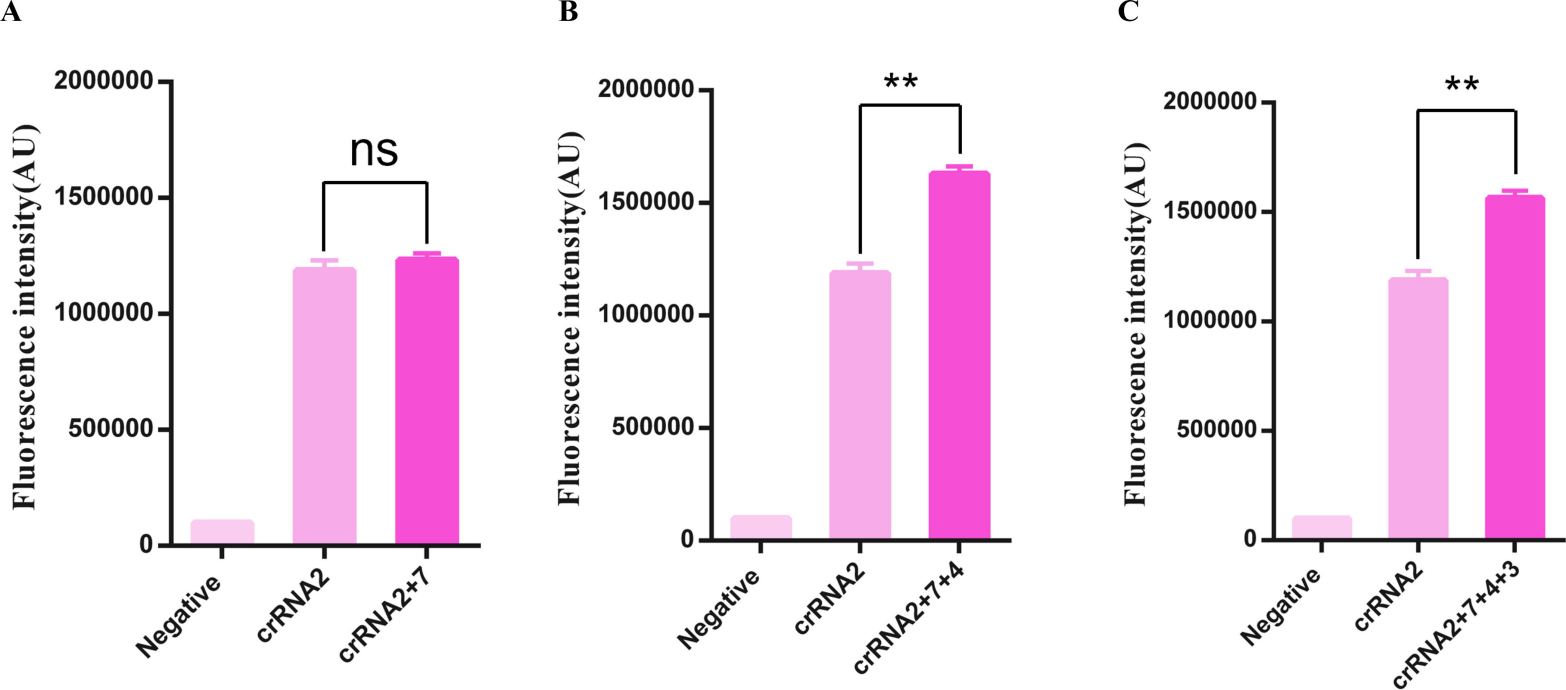
Screening of crRNA combinations. Comparison of the enhancement effect of fluorescence signals produced by the CRISPR/Cas12a system with single or multiple crRNAs. crRNAs are combined by using two crRNAs (crRNA 2 + crRNA 7), three crRNAs (crRNA 2 + crRNA 7 + crRNA 4), and four crRNAs (crRNA 2 + crRNA 7 + crRNA 4 + crRNA 3). (**A**) The fluorescence intensity of the combination of two crRNAs has no significant change with that of a single crRNA. (**B**) The combination of three crRNAs can significantly enhance the sensitivity of the CRISPR/Cas12a system compared with a single crRNA. (**C**) The combination of four crRNAs showed significant improvement. Data were compared by *t* tests (**P < 0.01).

Based on the previous efficiency test, the crRNA sensitivity ranking from highest to lowest is as follows: crRNA 2, crRNA 7, crRNA 4, crRNA 3, crRNA 9, crRNA 1, crRNA 10, crRNA 5, crRNA 6, and crRNA 8. Next, we combined the crRNAs by using two crRNAs (crRNA 2 + crRNA 7), three crRNAs (crRNA 2 + crRNA 7 + crRNA 4), and four crRNA (crRNA 2 + crRNA 7 + crRNA 4 + crRNA 3). The working concentration of Cas12a is set to 50 nM, and the final concentration of crRNA is set to 100 nM, which is consistent with the reaction amount used in a single crRNA system, and the concentration of Cas12a was evenly distributed among each crRNA. The CRISPR/Cas12a detection reaction with multiplex crRNA was conducted at 37°C for 90 min, followed by the detection of fluorescence signals. As shown in Fig. 3, the combination of crRNAs, especially a combination of three crRNAs and four crRNAs, can significantly enhance the sensitivity of the CRISPR/Cas12a system compared to a single crRNA. In comparison, the combination of three crRNAs (crRNA 2 + crRNA 7 + crRNA 4) showed the most significant improvement.

### Specificity and sensitivity evaluation

**Fig 4:**
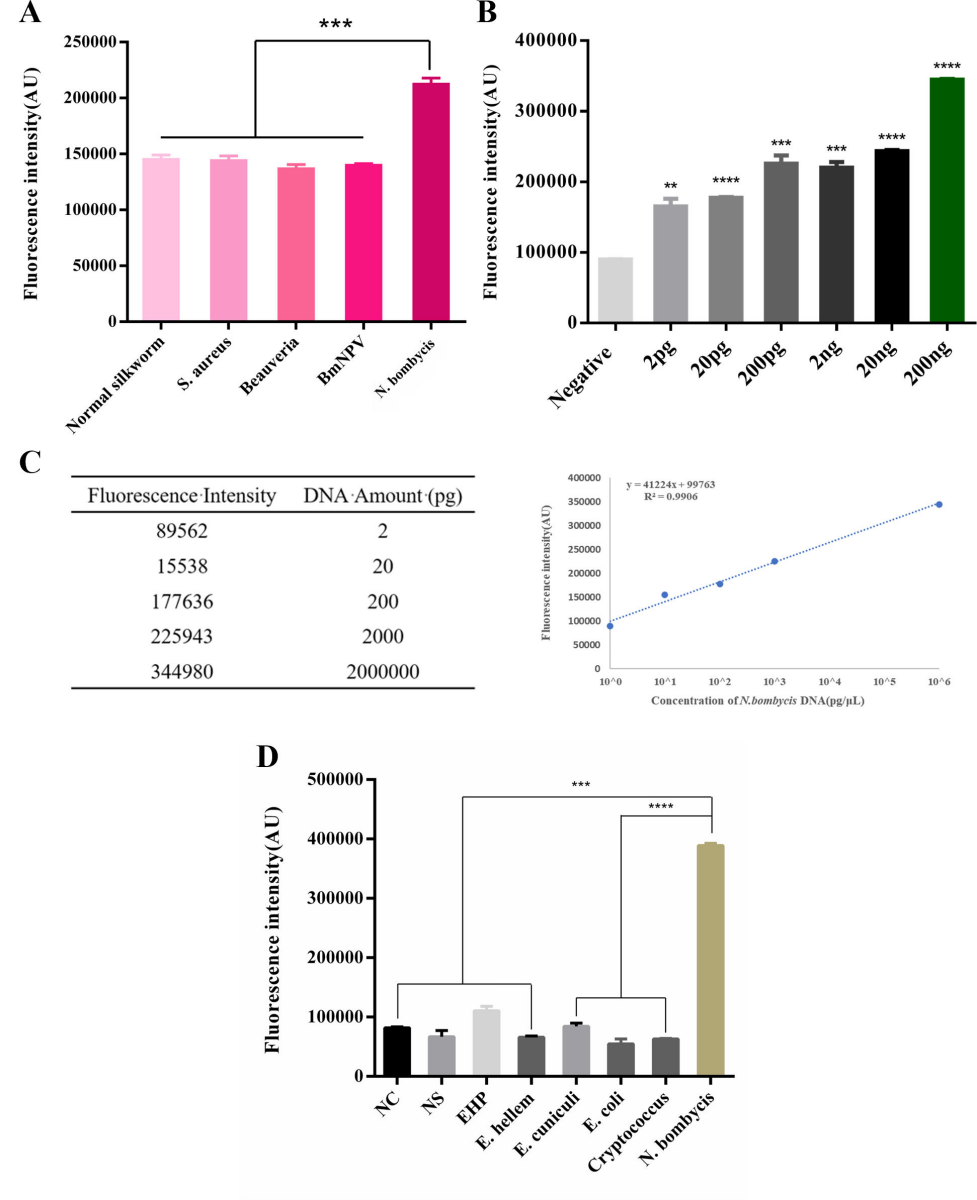
Sensitivity and specificity evaluation of the multiplex crRNA CRISPR/Cas12a nucleic acid amplification-free detection method. (**A**) Fluorescence intensity of different silkworm pathogens detected by the multiplex-crRNA CRISPR/Cas12a method. (**B**) The end-point fluorescence values of a dilution series of *N. bombycis* DNA. (**C**) Relationship between genome concentration of *N. bombycis* and fluorescence intensity. Data were compared by *t* tests. (**D**) Fluorescence intensity of other microsporidia and other pathogens detected by the multiplex-crRNA CRISPR/Cas12a method (NC: negative control, NS: normal control; **P < 0.01, ***P < 0.001, and ****P < 0.0001).

In the experiment evaluating the specificity of the detection system, the results showed that the terminal fluorescence intensity of the *N. bombycis* group in the multiplex-crRNA CRISPR/CAS12a system was significantly higher than that of other groups. The other groups showed similar fluorescence signals to the negative control group, indicating that the detection system had reasonable specificity (Fig. 4A). Next, we extracted genomic DNA from *N. bombycis*. We diluted it in a series to evaluate the sensitivity of the multiplex-crRNA CRISPR/Cas12a detection system. Following incubation at 37°C for 90 min, we collected fluorescence signals and analyzed the terminal fluorescence value of each sample. The results demonstrated that the multiplex-crRNA CRISPR/Cas12a method could detect the genomic DNA of *N. bombycis* as low as 2 pg (Fig. 4B). To verify whether the fluorescence signal generated by the detection is proportional to the concentration of the target DNA, we used various amounts of genomic DNA and calculated correlations between the detected fluorescence and the DNA amounts in the system. The results demonstrated that the fluorescent signals are proportional to the *N. bombycis* DNA amounts (Fig. 4C), indicating that the multiplex-crRNA CRISPR/Cas12a detection method had the potential for nucleic acid quantification. As a result, we may be able to use the formula to estimate the pathogen load in unknown samples directly. Moreover, due to different environments, we also added other microsporidia and other pathogens, such as *Encephalitozoon cuniculi*, *Encephalitozoon hellem*, *Enterocytozoon hepatopenaei* (EHP), *Escherichia coli*, and *Cryptococcus* for specific detection, not just silkworm pathogens. The results showed that the terminal fluorescence intensity of *N. bombycis* group in the Multiplex-crRNA CRISPR/CAS12a system was significantly higher than that of other groups, and the other groups showed similar fluorescence signals to the negative control group (Fig. 4D).

### Detection of infected silkworm midgut and blood samples

**Fig 5:**
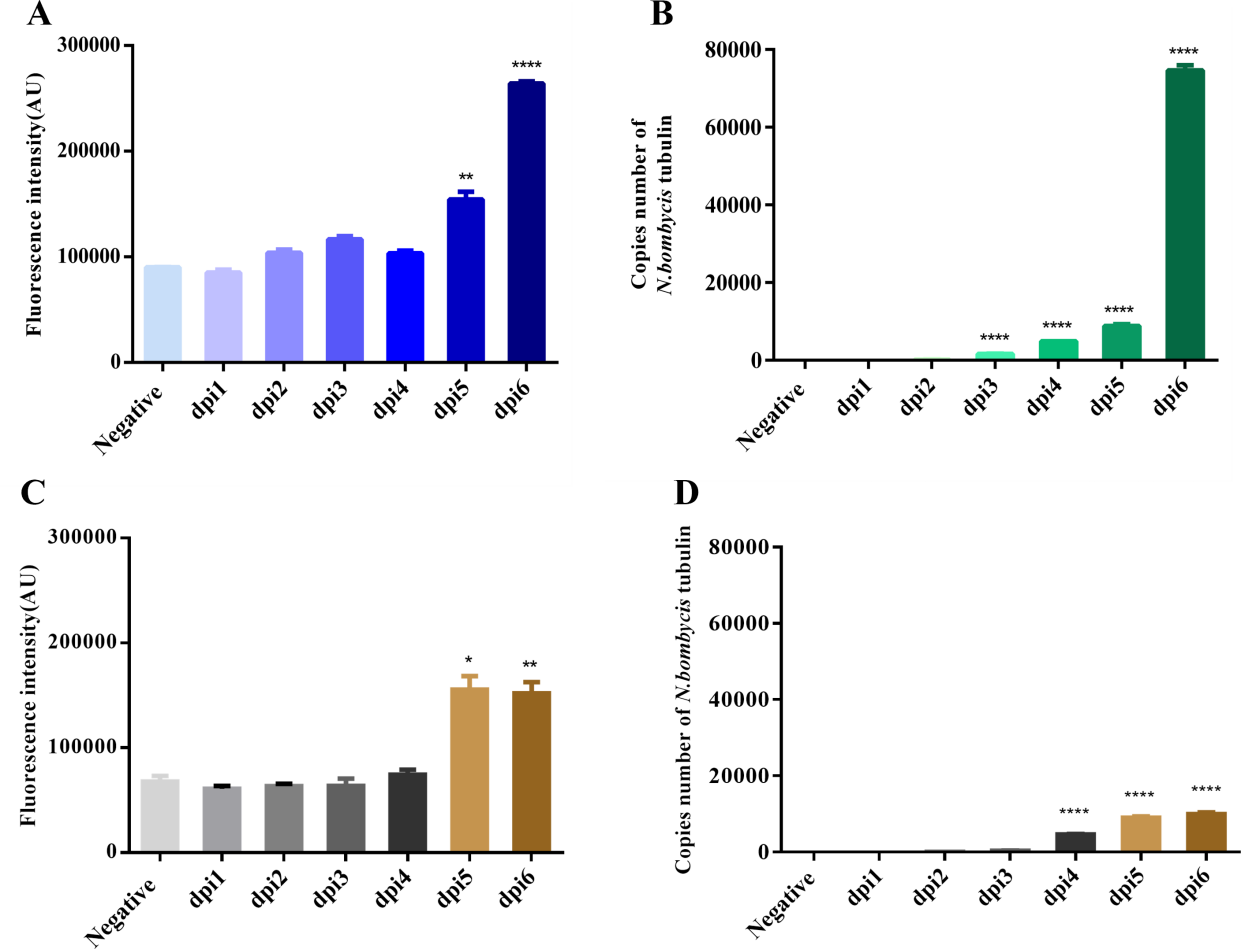
Detection of infected silkworm midgut and blood. (**A**) The end-point fluorescence signals of *N. bombycis*-infected silkworm midgut on different days. (**B**) qPCR result of *N. bombycis*-infected silkworm midgut on different days. (**C**) The end-point fluorescence signals of *N. bombycis*-infected silkworm blood on different days. (**D**) qPCR detection of *N. bombycis*-infected silkworm blood on different days. Data were compared by *t* tests (*P < 0.05, **P < 0.01, and ****P < 0.0001).

In this part of the work, we extracted DNA from the midgut and blood of infected silkworms and un-infected controls from 1 to 6 dpi. These DNA samples were used as templates for detection using this method. Considering the proportion of pathogenic genomes in the silkworm midgut tissue genome, the total amount of DNA was 10 µg for detection in this method. The detection showed positive results that the method can successfully detect the midgut as well as blood samples of infected silkworms (Fig. 5A and C). We also used qPCR for verifications of the existence of *N. bombycis* (Fig. 5B and D). Both methods could efficiently detect *N. bombycis*, especially from 3 dpi and on, significantly on 5 and 6 dpi. Taken together, the newly developed multiplex-crRNA CRISPR/Cas12a nucleic acid amplification-free detection method is capable of detecting *N. bombycis* in infected tissue samples.

## DISCUSSION

The CRISPR/Cas system becomes a powerful tool for disease and pathogen detection and diagnosis especially when combined with on-site real-time monitoring equipment. It has been developed and successfully applied to nucleic acid detection of various pathogens, including virus (18 – 22), bacteria (23 – 27), fungi (28 – 30), and parasites (31). However, many pathogen detection technologies based on the CRISPR/Cas12a system rely on nucleic acid isothermal amplification technology. While this amplification method offers high efficiency, it can only detect pathogens qualitatively, making it challenging to achieve quantitative detection. Moreover, introducing upstream and downstream primers in the reaction system can lead to false-positive or false-negative results, necessitating further verification and extending the overall detection time. As a result, there is ample room for improvement of detection technologies based on the CRISPR/Cas system.

Our newly developed multiplex-crRNA CRISPR/Cas12a detection system would solve the above issues. By designing multiplex crRNAs complementary to the same target sequence, multiple Cas12a-crRNA complexes target the same sequence, leading to the specific cleavage of the target DNA and nonspecifically cleaving any ssDNA in the system. Compared to a single crRNA system, using multiple crRNAs increases the cutting efficiencies upon single-stranded DNA probes and enhances the fluorescence signal without pre-amplification. As a result, many limitations associated with isothermal amplification are avoided, and since the multiplex-crRNA CRISPR/Cas12a detection system directly detects pathogens, nucleic acid amplification is unnecessary, enabling quantitative detection of pathogens. Our method could detect as low as 2 pg of pathogenic DNA, which would proximately equal to 2 × 10^2^ spores in the system (32). This is a feasible and sensitive detection method. To further improve the detection sensitivity of the amplification-free CRISPR/Cas technology, four main strategies could be pursued: optimizing key parameters of the CRISPR/Cas reaction, developing sensitive digital detection platforms, coupling with other sensitive signal sensors, and designing cascade reactions to achieve signal amplification (33). As we optimize our multiplex-crRNA CRISPR/Cas12a nucleic acid amplification-free detection method, we also hope to realize visual detection, which can be applied to future field detection.

### Conclusions

**Fig 6:**
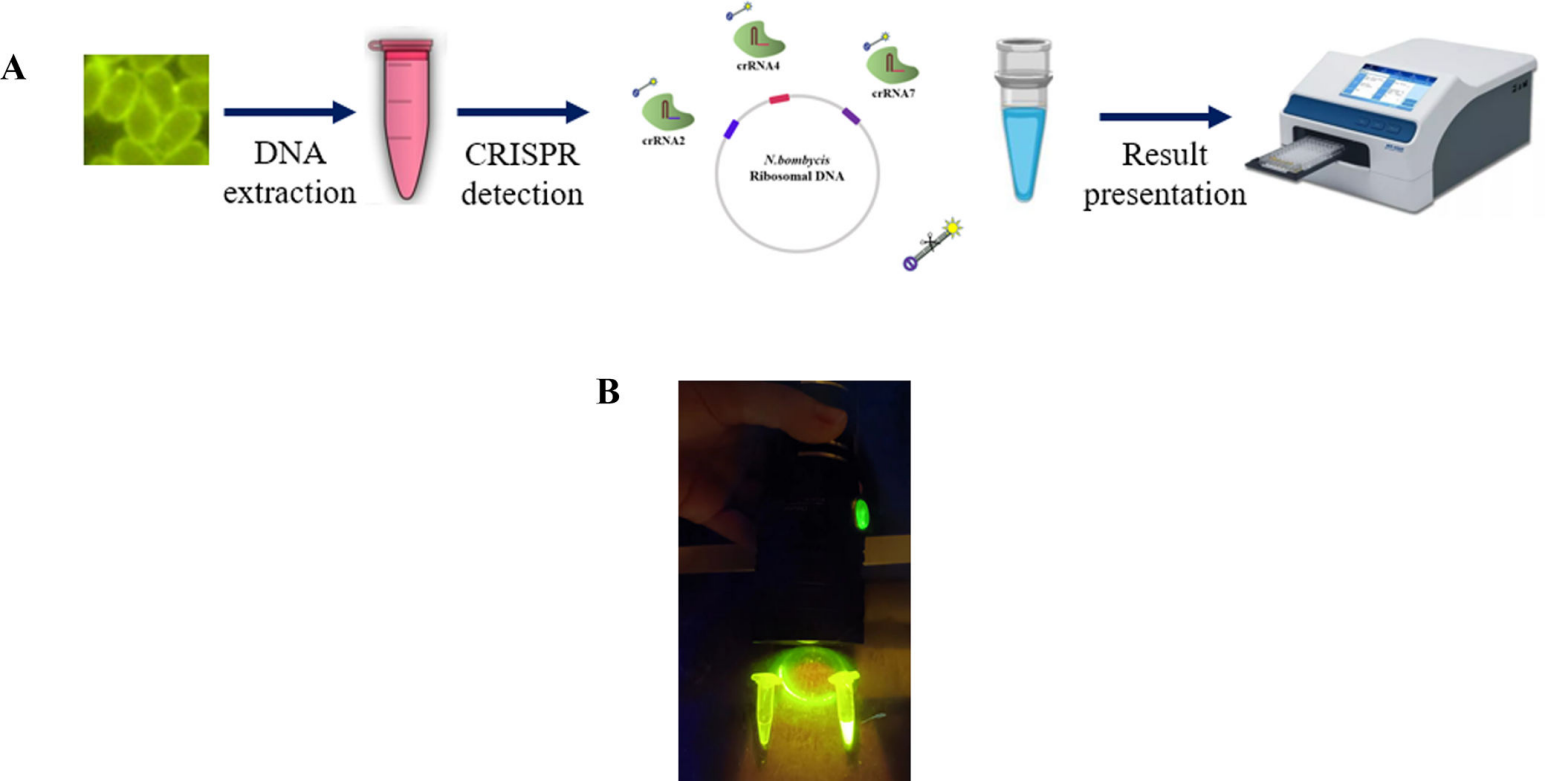
(**A**) Schematic illustration of the multiplex-crRNA CRISPR/Cas12a nucleic acid amplification-free detection method for *N. bombycis*. The process of detection is as follows: Genomic DNA from *N. bombycis* spores or *N.bombycis*-infected silkworms was extracted; the DNA sample was added to test tubes containing the multiplex-crRNA of CRISPR/Cas12a detection system; amplification-free reactions took place by incubation at 37°C for 90 min; and end-point fluorescence signals were detected by a plate reader or a blue light torch. (**B**) Display of blue-light torch on detection of multiplex-crRNA CRISPR/Cas12a amplification-free detection results. The fluorescence light could be directly observed by torchlight (right tube), while the negative control showed no signal (left tube). This demonstrates that our method is feasible, simple, and fast in the field usage.

In this study, we demonstrated that combining multiple crRNAs in the same reaction can significantly enhance the detection sensitivity of the CRISPR/Cas12a system, enabling amplification-free detection, which is a simple and rapid detection method that can be easily applied in the field (Fig. 6). Among the various combinations tested, the multiplex-crRNA CRISPR/Cas12a detection system containing three crRNAs showed the most potent synergistic effect. Based on these findings, we established an amplification-free method for the direct detection of *N. bombycis* (genomic DNA 2 pg, approximately 2 × 10^2^ spores is the lowest limit). This approach allows for quantitative detection of *N. bombycis* spores, enabling the direct calculation of pathogen load during infection. Notably, the method exhibits high specificity for *N. bombycis* and does not cross-react with *Staphylococcus aureus*, *Beauveria bassiana*, Bombyx mori nucleopolyhedrovirus, *E. cuniculi*, *E. hellem*, EHP, *E. coli*, and *Cryptococcus*. By eliminating the need for nucleic acid amplification, the multiplex-crRNA CRISPR/Cas12a detection method saves hands-on time, reduces the risk of aerosol pollution, and can be directly applied in field detection of *N. bombycis*. This study provides a new approach for the inspection and quarantine of silkworm pébrine disease in sericulture and provides a new method for the detection of other pathogens.

## MATERIALS AND METHODS

### *N. bombycis* extraction and purification

Fourth instar molted silkworm larvae were fed mulberry leaves smeared with *N. bombycis* spores (approximately 10^5^ spores/larva). Silkworm pupae were collected for the extraction and purification of *N. bombycis*. The silkworm chrysalis was ground into a homogenate in a mortar and a pestle, ddH_2_O was added to the homogenate, and the homogenate was filtered through gauze and collected. The filtrate was centrifuged to obtain a crude extract, which was subsequently subjected to Percoll (Cytiva, Sweden) density gradient centrifugation at 13,000 g for 30 min for fine purification. The bottom pellet of purified spores was collected.

### Preparation of infected silkworm samples

Fifth instar molted silkworm larvae were fed mulberry leaves smeared with *N. bombycis* spores (approximately 10^5^ spores/larva). Normal and infected silkworms were dissected, and the blood and midgut of silkworms were taken from 1 to 6 dpi.

### Extraction of genomic DNA from samples

The microsporidian spore (10^9^) was treated with cetyl trimethyl ammonium bromide (CTAB) buffer (100 mmol/L Tris-HCI, pH 8.0, 20 mmol/L EDTA, 1.4 mol/L NaCI, 0.2%–1% 2-hydroxy-1-ethanethiol) and proteinase K (20 mg/mL) at 60°C for 4 h, followed by the extraction of DNA by the CTAB method as described previously (34). The concentration of the extracted DNA sample was measured by NanoDrop (DeNovix, USA). It was applied to the establishment, specificity evaluation, and sensitivity evaluation of multiplex-crRNA CRISPR/Cas12a methods. Besides detecting infected silkworm blood and midgut, the CTAB method is also used to extract genomic DNA.

### Target DNA preparation and purification

The rDNA gene was cloned by PCR from *N. bombycis* genomic DNA and inserted into a pESI-Blunt simple vector (Yeasen, China). After successful sequencing, the screening experiment of crRNA was carried out.

### Design and synthesis of crRNA

The rDNA of *N. bombycis* is highly conservative and can be used to distinguish *N. bombycis* from other silkworm pathogens. Therefore, the rDNA sequence (AY259631.1) was utilized for designing crRNAs for *N. bombycis*. According to the requirements of the Cas detection system, the crRNA of CRISPR/Cas12a system consists of direct repeat sequences and spacer sequences. The natural repeat sequences of 21 bp (5′-UAAUUUCUACUAAGUGUAGAU-3′) form a hairpin structure, and the spacer sequences of 21 bp are complementary to the target DNA and are adjacent to a PAM sequence (13). The target sequence was put into the online design website CRISPOR (tefor.net) to select the target fragment with a high score, then the target fragment recommended by the website was put into NCBI for sequence comparison, and 10 target fragments with reasonable specificity were selected. The direct repeat and target fragment sequences constitute a complete crRNA sequence. Finally, it can be transcribed and synthesized *in vitro* by adding a T7 promoter sequence (5′-TAATACGACTCACTATAGGG-3′) before the designed crRNA sequence.

### Multiplex-crRNA CRISPR/Cas12a reaction system

The optimized CRISPR/Cas12a reaction solution contains 50 nM LbCas12a protein (Magigen, China), 100 nM crRNA, 1× reaction buffer (500 mM NaCl, 100 mM Tris-Tricine, pH 7.9, 100 mM MgCl_2_, and 10 mM DTT; Magigen, China), and 500 nM TTTTTTT ssDNA probe (5’FAM, 3’BHQ1; Sangon, China). The above concentration represents the final concentration in the reaction system. Next, 1 µg of target nucleic acid solution was added to the CRISPR reaction mixture, and the appropriate amount of water was added to make the total volume of the reaction system to 50 µL. After the CRISPR reaction mixture was incubated in an incubator at 37°C for 90 min, a multifunctional enzyme-labeled instrument read the fluorescence intensity at 535 nm under the excitation light of 485 nm wavelength.

### Evaluation of the specificity of the multiplex-crRNA CRISPR/Cas12a detection system

To verify the specificity of the developed multiplex crRNA strategy, we detected *N. bombycis* and other pathogens using the multiplex-crRNA CRISPR/Cas12a method. Other pathogens included *B. bassiana*, *S. aureus*, BmNPV, *E. cuniculi*, *E. hellem*, *E. coli*, EHP, and *Cryptococcus*. The normal control group was normal silkworm genomic DNA, and the template of the negative control group was enzyme-free water without other genomic DNA.

### Evaluation of the sensitivity of the multiplex-crRNA CRISPR/Cas12a detection system

Different numbers of *N. bombycis* genomes (2 pg, 20 pg, 200 pg, 2 ng, 20 ng, and 200 ng) were used as templates of the multiplex-crRNA CRISPR/Cas12a detection system to evaluate the sensitivity of the detection system. At the same time, a negative control was set up, and the data were statistically analyzed after completion.

## Data Availability

The data that support the findings of this study are available from the corresponding authors upon reasonable request. The rDNA sequence of *N. bombycis* was deposited in NCBI (AY259631.1).
